# Gut metagenomic analysis reveals prominent roles of *Lactobacillus* and cecal microbiota in chicken feed efficiency

**DOI:** 10.1038/srep45308

**Published:** 2017-03-28

**Authors:** Wei Yan, Congjiao Sun, Jingwei Yuan, Ning Yang

**Affiliations:** National Engineering Laboratory for Animal Breeding and MOA Key Laboratory of Animal Genetics and Breeding, College of Animal Science and Technology, China Agricultural University, Beijing, 100193 China

**Keywords:** Agricultural genetics, Applied microbiology

## Abstract

Interactions between the host and gut microbiota can affect gut metabolism. In this study, the individual performances of 252 hens were recorded to evaluate feed efficiency. Hens with contrasting feed efficiencies (14 birds per group) were selected to investigate their duodenal, cecal and fecal microbial composition by sequencing the 16S rRNA gene V4 region. The results showed that the microbial community in the cecum was quite different from those in the duodenum and feces. The highest biodiversity and all differentially abundant taxa between the different efficiency groups were observed in the cecal microbial community with false discovery rate (FDR) <0.05. Of these differentially abundant cecal microbes, *Lactobacillus* accounted for a greater proportion than the others. The abundances of *Lactobacillus* and *Akkermansia* were significantly higher while that of *Faecalibacterium* was lower (FDR < 0.05) in the better feed efficiency (BFE) group. Phylogenetic investigation of communities by reconstruction of unobserved states (PICRUSt) analysis revealed that the functions relating to glycometabolism and amino acid metabolism were enriched in the cecal microbiota of the BFE group. These results indicated the prominent role of cecal microbiota in the feed efficiency of chickens and suggested plausible uses of *Lactobacillus* to improve the feed efficiency of host.

**Supplementary information:**

The online version of this article (doi:10.1038/srep45308) contains supplementary material, which is available to authorized users.

## Introduction

The gastrointestinal tract is the major site of food digestion and nutrient absorption. Cecum is the chief functional section in the distal intestine, and its importance in birds’ metabolism has received increasing attention^1,2^. The cecum, which is full of microbial fermentations, plays important roles in preventing pathogen colonization, detoxifying harmful substances, recycling nitrogen and absorbing additional nutrients^3^. The digestibility and the ability to metabolize crude fiber or other nutrients are lower in birds with a cecectomy than in normal birds^4^. In addition, significant absorption of glucose was observed in the cecum^5^, and a higher ability to actively absorb sugars at low concentrations was found in the cecum compared with the jejunum^6^. Located at the beginning of the intestine, the duodenum is crucial for feed digestion and absorption; it has a lower pH than the hindgut and is the region that absorbs most glucose^7^ and other nutrients within the small intestine^8,9^.

Although the cecum and the duodenum themselves are important, interactions between the gut and commensal microbes may exert a significant influence on the function of the intestine. Previous studies showed that the digestion of uric acid, cellulose, starch and other resistant carbohydrates in the cecum was associated with the cecal microbial members^3,10,11^. In a recent study, numerous oligosaccharide- and polysaccharide-degrading enzyme-encoding genes and several pathways involved in the production of short-chain fatty acids (SCFAs) were observed in the cecal metagenome of the chicken^12^. The SCFAs were produced mainly by microbial fermentation in the hindgut and could be absorbed through the mucosa and catabolized for energy by the host^13^; the SCFAs also inhibited acid-sensitive pathogens by lowering the pH^14^. Due to the rapid flow of the highly fluid, digested material and a higher acidity, the number of microbes in the duodenum was lower than that in the posterior intestine. *Lactobacilli* and *Lactobacillaceae* were observed to be the predominant microbes in the duodenum of chickens^15^ and mice^16^, respectively. However, the relationship between the duodenal microbiota and the host nutritional metabolism is poorly understood. Feces have been widely used for metagenomic studies, because their easy to collection, allowing a continuous observation of the changes during a period without complicated operations or sacrifices, however, the microbial relationships between feces and intestinal segments in layer chickens are still unclear and need to be explored^15,16,17^.

For farm animals, great attention is paid to feed efficiency which is a comprehensive trait to evaluate the efficacy of nutrient and energy metabolism. Improving feed efficiency can decrease the cost to producers, preserve additional edible resources for humans, and reduce the excrement effluent and the emission of greenhouse gases. The feed conversion ratio (FCR) and residual feed intake (RFI) are the major indices for assessing the feed efficiency of animals. FCR has been used in breeding for a long time because of its convenience and effect on improving growth. However, in contrast to RFI, FCR does not include variability in the maintenance requirement for feed intake^18^ and does not distribute normally^19^. Koch *et al*.^20^ proposed the concept of RFI, which accounts for both maintenance requirements and growth. Because of its phenotypic independence from maintaining body weight and body weight gain, RFI has been proposed for measuring feed efficiency in breeding, with the heritability of RFI in chickens ranging from 0.2 to 0.8^21,22,23,24^. Feed efficiency is a complex trait because it is influenced not only by the host genetics and physiological state but also by the intestinal microbiota, which would affect the nutrient digestion and energy absorption of the host. Singh *et al*.^25^ investigated the difference in microbial communities between good and poor feed efficiency broilers using fecal samples; *Acinetobacter, Anaerosporobacter* and *Arcobacter* were dominant in the poor efficiency group, whereas *Escherichia/Shigella, Faecalibacterium* and *Helicobacter* were dominant in the better efficiency group. However, the abundances of *Lactobacillus* and *Bacteroides* were similar in both groups. Mignon-Grasteau *et al*.^26^ quantified the microbial 16S rDNA in the cecum by qPCR and observed higher ratios of *Clostridium leptum, Clostridium coccoide*s and *Lactobacillus salivarius* to *E. coli* in the better efficiency group. Nevertheless, the feed efficiency in both studies was represented by FCR, and the relationships between RFI and the gut microbiota remain to be understood.

Next-generation sequencing techniques have been used to study microbiota composition and extend the understanding of the interactions between the host and commensal microbes in feed efficiency studies. However, the microbial communities have not been compared among the foregut (duodenum), hindgut (cecum) and feces in hens, and the interactions between the feed efficiency evaluated using RFI and gut microbiota need to be explored.

## Results

### Phenotypic and sequencing data

The daily feed intake (FI), daily egg mass (EM), average body weight (BW) and residual feed intake (RFI) at 32–44 (T1) and 57–60 (T2) weeks of age are listed in Table 1. The RFI value of the better feed efficiency (BFE) group was found to be significantly lower (*P* < 0.01) than that of the poor feed efficiency (PFE) group. The FIs of the BFE group were 17.0 and 24.3 percent lower than those of the PFE group in T1 and T2, respectively. No significant difference was found in the EM and BW between the two groups.

**Table 1:** Summary of phenotypes in the two laying periods.

	32–44 wks		57–60 wks	
	BFE	PFE	BFE	PFE
Average daily feed intake (g)	80.8 ± 12.3^A^	97.4 ± 4.1^B^	84.7 ± 16.4^A^	111.9 ± 17.4^B^
Average daily egg mass (g)	36.0 ± 8.7	40.0 ± 3.7	40.3 ± 6.6	32.9 ± 12.5
Average body weight (g)	1258.0 ± 150.2	1313.6 ± 90.7	1393.4 ± 97.3	1402.3 ± 108.3
RFI value	−5.66 ± 2.26^A^	6.18 ± 2.15^B^	−10.06 ± 2.64^A^	14.69 ± 4.34^B^

Different superscripted capital letters in the same period indicate a significant difference at *P* < 0.01. BFE and PFE denote better feed efficiency and poor feed efficiency, respectively.

The 16S rRNA gene-based sequencing produced millions of raw reads. After assembly and filtration, the BFE samples had an average of 44,998, 102,993 and 66,094 clean tags in the duodenum, cecum and feces, respectively. In addition, the clean tags for the PFE samples in the duodenum, cecum and feces were 37,404, 91,143 and 65,315, respectively. The average length of the clean tags was 253 bp, and the tags were taxonomically classified from kingdom to species.

### Predominant microbes

The predominant microbes in the duodenum, cecum and feces were similar at the phylum level, in which *Firmicutes* and *Bacteroidetes* were the major microbes. However, the relative abundances of these two phyla were quantitatively different among the three sites (Table 2). *Firmicutes* accounted for more than 50% of the duodenal and fecal community, while only approximately 26% was observed in the cecal community. In contrast, greater than 50% *Bacteroidetes* was observed in the cecal microbial community, while less than 20% was found in both duodenum and feces. In the BFE group, the *Firmicutes* in the duodenum and *Verrucomicrobia* in the cecum were more abundant (*P* < 0.01), while the *Fusobacteria* in duodenum was less abundant (*P* < 0.01) than in the PFE group (Table 2).

**Table 2:** Relative abundance of the dominant phyla in duodenum, cecum and feces of the laying hens.

Duodenum (%)				Cecum (%)				Feces (%)			
Kingdom	Phylum	BFE^1^	PFE	Kingdom	Phylum	BFE	PFE	Kingdom	Phylum	BFE	PFE
Bacteria	Firmicutes	76.52^**A**^	65.18^**B**^	Bacteria	Bacteroidetes	54.86	56.28	Bacteria	Firmicutes	54.57	61.41
Bacteria	Bacteroidetes	7.10	12.73	Bacteria	Firmicutes	27.74	26.93	Bacteria	Bacteroidetes	15.84	13.77
Bacteria	Proteobacteria	7.41	8.35	Bacteria	Proteobacteria	6.02	7.16	Bacteria	Fusobacteria	8.93	14.19
Bacteria	Fusobacteria	0.93^**A**^	6.39^**B**^	Bacteria	Fusobacteria	2.91	1.62	Bacteria	Proteobacteria	15.25	6.55
Bacteria	Cyanobacteria	2.01	2.60	Bacteria	Cyanobacteria	0.53	0.50	Bacteria	Cyanobacteria	0.48	0.55
Archaea	Euryarchaeota	2.13	1.10	Bacteria	Tenericutes	0.42	0.45	Bacteria	Actinobacteria	0.35	0.23
Bacteria	Acidobacteria	0.76	0.36	Bacteria	Verrucomicrobia	0.52^**A**^	0.04^**B**^	Bacteria	Tenericutes	0.30	0.22
Bacteria	Actinobacteria	0.70	0.39	Bacteria	Deferribacteres	0.13	0.20	Bacteria	Acidobacteria	0.05	0.08
Bacteria	Verrucomicrobia	0.41	0.24	Bacteria	Spirochaetes	0.19	0.12	Bacteria	Verrucomicrobia	0.04	0.08
Bacteria	Planctomycetes	0.33	0.20	Bacteria	Actinobacteria	0.14	0.13	Archaea	Euryarchaeota	0.07	0.03

Different superscripted capital letters in the same segment indicate an adjusted significant difference at *P* < 0.01. BFE and PFE denote better feed efficiency and poor feed efficiency, respectively.

**Figure 1:**
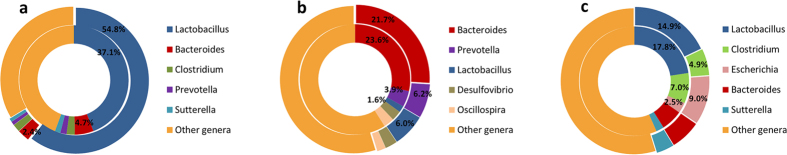
Predominant microbes in (**a**) duodenum, (**b**) cecum and (**c**) feces at the genus level. The outer and inner rings indicate the better feed efficiency and poor feed efficiency groups, respectively.

At the genus level, the top five abundant genera are shown in Fig. 1. *Lactobacillus* (54.8% in BFE, 37.1% in PFE), followed by *Bacteroides* (2.4% in BFE, 4.7% in PFE), was predominant in the duodenum. The cecum was dominated by *Bacteroides* (21.7% in BFE, 23.6% in PFE), followed by *Prevotella* (6.2% in BFE, 3.9% in PFE). In addition, the feces were dominated by *Lactobacillus* (14.9% in BFE, 17.8% in PFE), followed by *Clostridium* (4.9% in BFE, 7.0% in PFE). The results suggested that the dominant microbes in the duodenum and feces had greater similarity than those in the cecum.

### Microbial diversity

**Figure 2:**
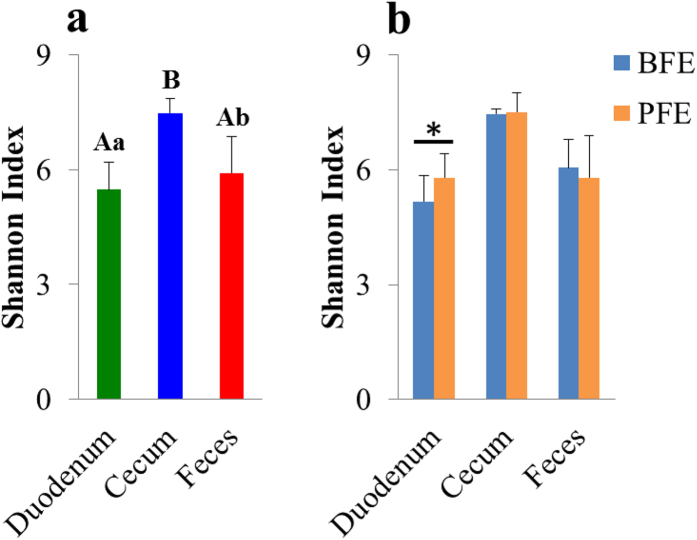
Shannon index in duodenum, cecum and feces and the contrasting feed efficiency groups. BFE and PFE denote better feed efficiency and poor feed efficiency, respectively. (**a**,**b**) Different superscripted small letters indicate significant difference at *P* < 0.05. (**a**,**b**) Different superscripted capital letters indicate significant differences at *P* < 0.01.

**Figure 3:**
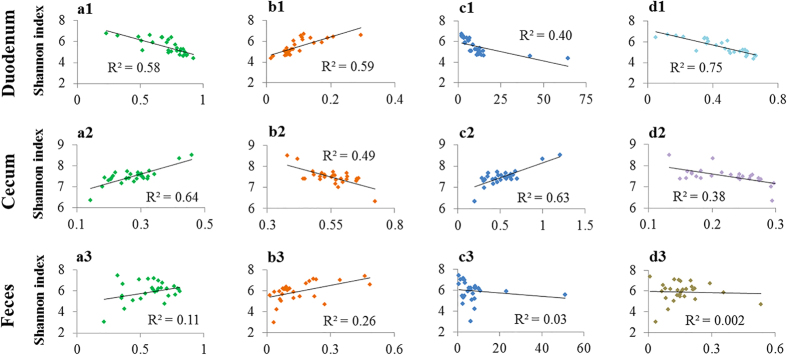
Regression curves. The vertical axes represent the Shannon index of the corresponding gut segments, and the horizontal axes represent the relative abundance of the corresponding microbes: **a1**–**a3** *Firmicutes*, **b1**–**b3** *Bacteroidetes*, **c1**–**c3** the ratio of *Firmicutes* to *Bacteroidetes* and d1–d3 *Lactobacillus.*

The Shannon index was used to evaluate the microbial community diversity. The cecal microbial community had higher diversity (*P* < 0.01) than the other two sites (Fig. 2a). Compared with the PFE group, the BFE group had significantly lower microbial diversity (*P* < 0.05) in the duodenum (Fig. 2b). Moreover, the Shannon index was correlated with the relative abundances of some microorganisms (Fig. 3). However, the correlation trends were quite different between the duodenum and the cecum. The Shannon index was negatively correlated with the relative abundance of *Firmicutes* (R^2^ = 0.58), positively correlated with *Bacteroidetes* (R^2^ = 0.59) and negatively correlated with the ratio of *Firmicutes* to *Bacteroidetes* (R^2^ = 0.40). In contrast, the cecal Shannon index was positively correlated *Firmicutes* (R^2^ = 0.64), negatively correlated with *Bacteroidetes* (R^2^ = 0.49) and positively correlated with the ratio of *Firmicutes* to *Bacteroidetes* (R^2^ = 0.63). However, these relationships in the feces were weak. At the genus level, a strong negative correlation between the Shannon index and the duodenal *Lactobacillus* was observed. However, the *Lactobacillus* in the cecum and feces was weakly correlated with corresponding Shannon index.

### Similarities of the microbial communities among the duodenum, cecum and feces

**Figure 4:**
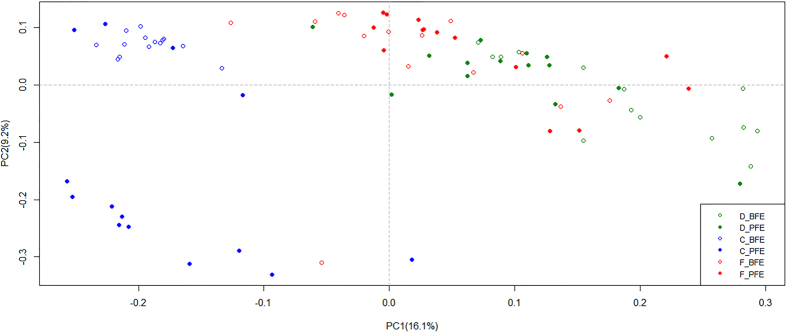
Principal coordinate analysis plot. BFE and PFE denote better feed efficiency and poor feed efficiency, respectively. “D_”, “C_” and “F_” represent the duodenum, cecum and feces, respectively.

Principal coordinate analysis (PCoA) and analysis of similarities (ANOSIM) were performed to compare the microbial similarities among the duodenum, cecum and feces. The visual plot created from the unweighted PCoA (Fig. 4) shows the relationships among the three sites. In addition, the ANOSIM numerically demonstrated that the duodenal microbial community was quite different from the cecal community (R = 0.96) but closer to the fecal community (R = 0.48). In addition, the observed difference between the BFE and PFE groups was greater (R = 0.35) in the cecum than in the duodenum (R = 0.18) and feces (R = 0.03) (Table 3).

**Table 3:** ANOSIM analysis results comparing duodenum, cecum and feces and between the different feed efficiency groups.

Comparison	R	*P*-values
Duodenum Vs. Cecum	0.96	<0.01
Duodenum Vs. Feces	0.48	<0.01
Cecum Vs. Feces	0.78	<0.01
D_BFE Vs. D_PFE	0.18	<0.01
C_BFE Vs. C_PFE	0.35	<0.01
F_BFE Vs. F_PFE	0.03	0.28

“R” is the index of ANOSIM that indicates the similarity of comparison group pairs. “R” ranges from −1 to 1: the pairs are more similar when the R index is closer to 0 and the pairs are different from each other when the R index is close to 1.

### Abundance differences in the microbiota between the BFE and PFE groups

**Figure 5:**
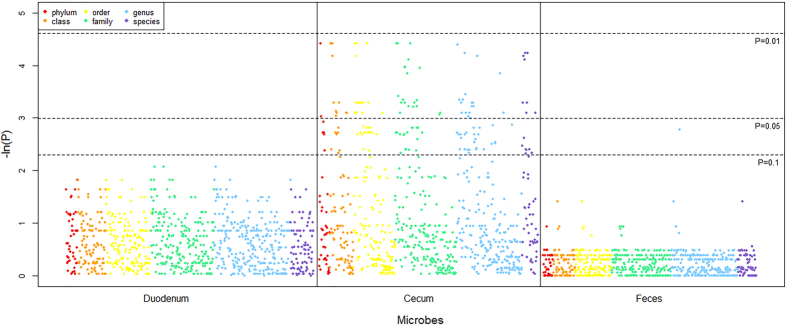
Negative logarithm scatter plot of the adjusted *P* values. The plot indicates -ln (adjusted *P*-values) (y-axis) plotted against all taxonomic microbes (x-axis) and the horizontal dotted lines depict the significant thresholds. The adjusted *P*-values have been corrected by FDR.

**Figure 6:**
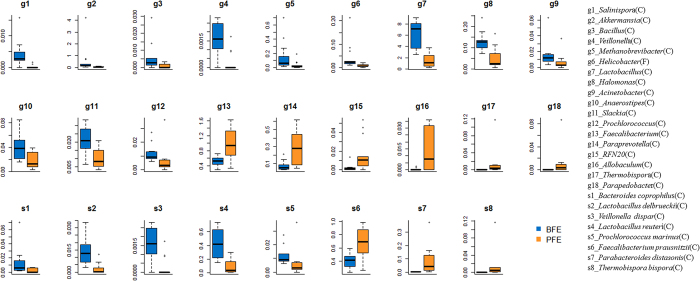
Box plots of differentially abundant genera and species in cecum (FDR < 0.05) and feces (FDR < 0.1). BFE and PFE denote better feed efficiency and poor feed efficiency, respectively.

To investigate the differences in microbial abundance between the contrasting feed efficiency groups, Mann-Whitney tests between the two groups were performed, and the negative logarithms of the false discovery rate (FDR) values are shown in Fig. 5. It is clear that all significantly different (FDR < 0.05) taxa were present in cecum. The significantly different taxa in the cecum (FDR < 0.05) and suggestively different taxa in the feces (FDR < 0.1) are listed in Supplementary Table. Of these taxa, 12 genera were more abundant in the BFE group than in the PFE group, while 7 genera were more abundantly in the PFE group (Fig. 6). Notably, there were significantly higher proportions of *Lactobacillus* and *Akkermansia* (FDR < 0.05) in the BFE group. The comparison also revealed the difference at the species level. The relative abundance of 5 species, *Bacteroides coprophilus, Lactobacillus delbrueckii, Veillonella dispar, Lactobacillus reuteri* and *Prochlorococcus marinus*, were significantly higher in the BFE group (FDR < 0.05), whereas 3 species, *Faecalibacterium prausnitzii, Parabacteroides distasonis* and *Thermobispora bispora*, were found to be significantly higher in the PFE group (FDR < 0.05).

### Prediction of gut microflora functions

**Figure 7:**
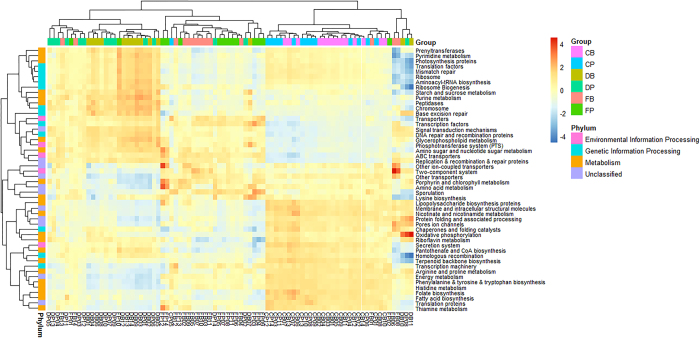
Heat map of the top 50 predicted functions by KEGG. CB, DB and FB denote the better feed efficiency groups in cecum, duodenum and feces, respectively. CP, DP and FP denote the poor feed efficiency groups in cecum, duodenum and feces, respectively.

**Figure 8:**
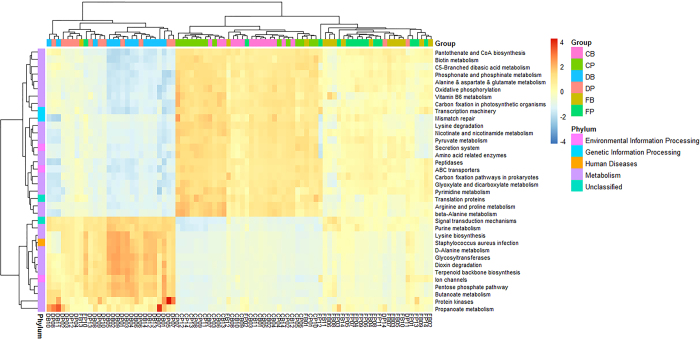
Heat map showing the different abundances of functions predicted by KEGG among duodenum, cecum and feces. CB, DB and FB denote better feed efficiency groups in cecum, duodenum and feces, respectively. CP, DP and FP denote poor feed efficiency groups in cecum, duodenum and feces, respectively.

Phylogenetic investigation of communities by reconstruction of unobserved states (PICRUSt) analysis was performed to predict microbial functions using the Kyoto Encyclopedia of Genes and Genomes (KEGG) and Clusters of Orthologous Groups of proteins (COGs) databases. The top 50 predicted functions were used as variants to hierarchically cluster the samples at the three sites (Fig. 7); this clustering showed the distinct functions of the metagenome in the duodenum, cecum and feces. Specifically, the functions associated with metabolism were mostly found in the cecum, followed by the duodenum. Additionally, the functions relating to genetic information processing were more frequent in the duodenum, while more unclassified functions were found in the feces. We also used the functions with statistically significant difference among the three sites to draw a hierarchical cluster plot (Fig. 8). This plot showed that most of the different functions were associated with the metabolism of nutrients, such as biotin, vitamin B6, pyruvate, butanoate and propanoate. Additionally, the functional profiles in duodenum and cecum exhibited opposite features while the profile in the feces was ambiguous.

**Figure 9:**
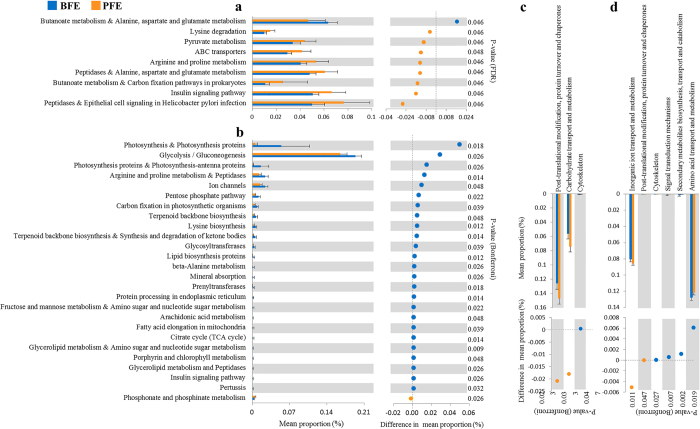
Differences in the abundance of KEGG and COG functions between the better feed efficiency and poor feed efficiency groups. BFE and PFE denote better feed efficiency and poor feed efficiency, respectively.

In the duodenum, the most significantly different functions (FDR < 0.05) between the contrasting feed efficiency groups were associated with protein and amino acid metabolism (Fig. 9a and c). Notably, a potential harmful function relating to epithelial cell signaling in *Helicobacter pylori* infection was enriched in the duodenum of the PFE group. In the cecum, the most significantly different functions, such as the functions related to photosynthesis, glycometabolism, ion transportation and amino acid metabolism, were enriched in the BFE group compared with the PFE group (Fig. 9b and d). In feces, no function was significantly different between the two groups.

## Discussion

Alterations in intestinal microbiota have been reported to have roles in affecting host metabolism^27,28^ and immune functions^29,30^. The finding that all differentially abundant (FDR < 0.05) microbes were observed in the cecum in the current study might have revealed a prominent role for cecal microbiota in feed efficiency. The microbial differences in the cecum might be: 1) the consequence of the physiological differences of the differential efficiency; 2) one of the factors influencing feed efficiency; or 3) the consequence of interactions with the host, which leads to the different feed efficiencies. The third possibility is likely the most acceptable one. At first, host genes shape the physiological environments as the “substrates” for microbes and the variations of “substrates” influence the gut microbial composition; then the metabolisms of gut microbiome affect the “substrates” in turn as feedback; finally, the interactions shape the host phenotype together^31,32^.

The cecum has been easily overlooked because of its location at the posterior segment of the intestine. With the increasing understanding from systems biology and high-throughput sequencing technology, the cecum and its microbiota are receiving growing attention in terms of disease^33,34^ and metabolism^35,36^. This study determined the microbial community composition and the predicted functions of the metagenome in the cecum, duodenum and feces and has thus provided comparative information for understanding the cecal microbiota.

However, the relative abundance of some differentially abundant microbes between the BFE and PFE groups was not consistent in the duodenum, cecum and feces. For example, the relative abundance of *Helicobacter* was found to be suggestively higher in the cecum of the PFE group (FDR = 0.073) but suggestively higher in the feces of the BFE group (FDR = 0.062). Therefore, caution should be taken when employing changes in the microbial community in feces as biomarkers to infer the state of the intestinal microbiota.

*Lactobacillus* was highly related with the host feed efficiency. *Lactobacillus* was one of the differentially abundant taxa and accounted for a greater proportion than did the other differentially abundant taxa. This genus, which is a beneficial commensal for humans and animals, has been studied and used in medicine and the food industry for years. Compared with the PFE group, the BFE group showed increases in duodenal *Lactobacillus (P* = 0.002, FDR = 0.162) and cecal *Lactobacillus delbrueckii* and *Lactobacillus reuteri* (FDR < 0.05) in the current study. Previous studies suggested that some species of *Lactobacillus* are associated with weight gain in human and animal infants^37,38,39^. Nevertheless, the body weight of the BFE group was not significantly higher than that of the PFE group, which was not consistent with the statement by Million^40^ that *Lactobacillus* would lead to obesity or weight gain. The results we obtained were in agreement with Lahtinen^41^, who suggested that some species of *Lactobacillus* would be associated with weight gain in infancy but not in human and animal adults. Although the effects of the enriched *Lactobacillus* might be different in infancy and adulthood, it could be inferred that the enriched *Lactobacillus* could generally improve the gastrointestinal tract and thus protect the gut from pathogens and promote efficient nutrient and energy extraction in the host.

Nevertheless, the increase in *Lactobacillus* would reduce the diversity of the microbial community in the corresponding gut segments, as shown in this study. Biodiversity is useful and important in indicating the health, disease and stability of ecosystems. An increase in microbial diversity in gut has been linked to improved health in the elderly^42^, while a loss of diversity has been associated with worsening of inflammatory bowel diseases (IBDs) and adiposity-related inflammation^43,44^. In another study, the gut microbial diversities of athletes were found to be significantly higher than those of the control groups^45^. Hence, even with a better feed efficiency, the decrease in diversity in the duodenal microbiota community in the BFE group might be a signal for some latent dangers due to a community imbalance. However, consistent results have not been observed in feces, suggesting that the diversity in feces should be cautiously used to represent the diversity of intestinal segments.

*Akkermansia spp.,* a widely studied microorganism that is inversely associated with obesity^46,47^, was found to be more abundant in the cecum of the BFE group. *Akkermansia* has been reported to be a mucin degradation-specialized bacterium that utilizes mucus as a sole carbon and nitrogen source^48^. An increase in *Akkermansia* has been shown to protect the niche from IBDs^49^, obesity^46,50^, and type I and type II diabetes mellitus^51,52^.

Interestingly, a potentially beneficial microbe, *Faecalibacterium prausnitzii*, was more abundant in the PFE group. This species has been found to be strongly reduced in the intestinal mucosa and fecal samples of patients with Crohn’s disease^53,54^. Because of the anti-inflammatory abilities of *F. prausnitzii*, the flourishing of this species in the PFE group might improve the ability of the host to protect against pathogens while consume more nutrients and energy as cost.

In the cecum, *Lactobacillus* accounted for approximately 4% of all microbes, but the relative abundances of other differentially abundant microbes accounted for not more than 1%. Although the relative abundances of these microbes were low in the microbial communities, they might “work” together to form a core measurable microbiota group that interacts with the host^55^; this possibility is similar to the polygene hypothesis in which proposes that many minor genes and several major genes (sometimes) are involved in the control of a quantitative trait. Hence, in this study, the *Lactobacillus* in the duodenum and cecum might play a “major gene” role, and other differentially abundant microbes in cecum perform the “minor gene” role in influencing host feed efficiency.

In conclusion, with the high-throughput sequencing technology, this study profiled the microbial communities in the duodenum, cecum and feces, which represent the niches of the anterior segment, posterior segment and the end of gastrointestinal tract, respectively. We found that the cecal microbiota was highly related to the feed efficiency, suggesting a prominent role for the cecal microbiota in chicken feed efficiency. The differentially abundant microbes, particularly *Lactobacillus*, might play a major role in affecting the feed efficiency. In addition, the differences among the three sites suggested that the fecal samples could be measured as references for the intestinal segments but could not reflect the actual status of the microbiota in the gastrointestinal tract. Therefore, the results provided a promising strategy to improve feed efficiency by cecal-oriented and differential microbiota-oriented alterations, and indicated that some segments (e.g., the cecum, which has not been well considered to date), should receive more attention for strategies to improve the health and nutrition of the host.

## Methods

### Animals and phenotypic data collection

The complete procedure was performed according to the regulations and guidelines established by the Animal Care and Use Committee of China Agricultural University. The entire study was approved by the committee (permit number: SYXK 2007–0023).

A line of brown-egg dwarf layer (DW), which has been maintained and selected mainly for egg production for more than 10 years in the Poultry Genetic Resource and Breeding Experimental Unit of China Agricultural University^56^, was used in this study. Two hundred and fifty two hens were randomly selected from a large population of this line and housed in individual cages in the same barn with *ad libitum* access to a layer diet. The cages allowed automatic recording for the egg production and feed intake every day from the 32^th^ to the 44^th^ week and from the 57^th^ to the 60^th^ week of age. The body weight was measured every 4 weeks^57^. The theoretical FI value was calculated using a lm procedure in an R project following the model^57^ of FI(expected) = b_0_ + b_1_MBW^0.75^ + b_2_EMDc + b_3_BWG, in which “FI(expected)”, “MBW”, “MBW^0.75^”, “EMDc” and “BWG” represent the expected feed intake, mean body weight, metabolic body weight, corrected egg mass production(adjusted abnormal egg) and body weight gain, respectively. RFI was then calculated from the actual FI by subtracting the expected FI. The RFI value, which was used to assess the feed efficiency, was negatively correlated with the feed efficiency.

### Sample collection, DNA extraction and sequencing of the 16S rRNA genes

Hens were ranked by RFI, after which the 14 hens with the lowest RFI and the 14 hens with the highest RFI were selected for sampling by the 60^th^ week of age. Fresh feces were collected from the 28 hens, after which the hens were humanly euthanized for collecting the duodenal and cecal contents. All samples were immediately placed in liquid nitrogen and stored at −80 °C.

Microbial genome DNA was extracted from the duodenal, cecal and fecal samples using a QIAamp DNA stool mini kit (QIAGEN, cat#51504)^58^ following the manufacturer’s instructions. The V4 hypervariable region of the 16S rRNA genes was PCR amplified from the microbial genomic DNA using primers 515F – 806R (515F: GTGCCAGCMGCCGCGGTAA, 806R: GGACTACHVGGGTWTCTAAT). All PCR reactions were performed in 30 μL reactions with 15 μL of Phusion^®^ High-Fidelity PCR Master Mix (New England Biolabs), 0.2 μM forward and reverse primers and approximately 10 ng of template DNA. Thermal cycling consisted of initial denaturation at 98 °C for 1 min, followed by 30 cycles of denaturation at 98 °C for 10 s, annealing at 50 °C for 30 s, and elongation at 72 °C for 30 s, followed by 72 °C for 5 min. Sequencing libraries were generated using the NEB Next^®^ UltraTM DNA library Prep Kit for Illumina (NEB, USA) following the manufacturer’s recommendations, and index codes were added. The library quality was assessed on a Qubit@ 2.0 Fluorometer (Thermo Scientific) and Agilent Bioanalyzer 2100 system (Agilent Technologies, Inc.). Finally, the library was sequenced on an Illumina Miseq platform and 2 bp/300 bp paired-end reads were generated.

### Data analysis

Paired-end reads from the original DNA fragments were merged using FLASH^59^ and were assigned to each sample. Sequences were analyzed using the QIIME^60^ software package, and those with ≥97% similarity were assigned to the same operational taxonomic units (OTUs). We pick a representative sequences for each OUT and use the Ribosomal Database Project (RDP) classifier to obtain taxonomic information for each representative sequence. Prediction of the microbial function was performed with PICRUSt^61^.

The data for the relative abundance of OTUs and predicted function were analyzed for statistical significance with the Mann-Whitney U test in R. *P*-values were adjusted by FDR using the BH method with the mt.rawp2adjp function in R (http://faculty.mssm.edu/gey01/-multtest/multtest-manual.pdf). ANOSIM analysis^62^ was performed in R with the package “vegan”. Principal coordinate analysis (PCoA) was conducted using QIIME.

## Additional Information

**How to cite this article**: Yan, W. *et al*. Gut metagenomic analysis reveals prominent roles of *Lactobacillus* and cecal microbiota in chicken feed efficiency. *Sci. Rep.* **7**, 45308; doi: 10.1038/srep45308 (2017).

**Publisher's note:** Springer Nature remains neutral with regard to jurisdictional claims in published maps and institutional affiliations.

## Supplementary information

Supplementary Dataset (XLS 41 kb)

Supplementary Information (PDF 663 kb)
